# Conjoint Analysis of Genome-Wide lncRNA and mRNA Expression during the Salicylic Acid Response in *Populus × euramericana*

**DOI:** 10.3390/plants12061377

**Published:** 2023-03-20

**Authors:** Chao Zhang, Yan Dong, Yachao Ren, Shijie Wang, Minsheng Yang

**Affiliations:** 1 Forest Department, Forestry College, Hebei Agricultural University, Baoding 071000, China; 2 Hebei Key Laboratory for Tree Genetic Resources and Forest Protection, Baoding 071000, China

**Keywords:** salicylic acid, *Populus × euramericana*, lncRNA–mRNA, plant stress resistance, plant disease resistance, growth and development

## Abstract

Long noncoding RNAs (lncRNAs) participate in a wide range of biological processes, but lncRNAs in plants remain largely unknown; in particular, we lack a systematic identification of plant lncRNAs involved in hormone responses. To explore the molecular mechanism of the response of poplar to salicylic acid (SA), the changes in protective enzymes, which are closely related to plant resistance induced by exogenous SA, were studied, and the expression of mRNA and lncRNA were determined by high-throughput RNA sequencing. The results showed that the activities of phenylalanine ammonia lyase (PAL) and polyphenol oxidase (PPO), in the leaves of *Populus × euramericana*, were significantly increased by exogenous SA application. High-throughput RNA sequencing showed that 26,366 genes and 5690 lncRNAs were detected under the different treatment conditions: SA and H_2_O application. Among these, 606 genes and 49 lncRNAs were differentially expressed. According to target prediction, lncRNAs and target genes involved in light response, stress response, plant disease resistance, and growth and development, were differentially expressed in SA-treated leaves. Interaction analysis showed that lncRNA–mRNA interactions, following exogenous SA, were involved in the response of poplar leaves to the external environment. Our study provides a comprehensive view of *Populus × euramericana* lncRNAs and offers insights into the potential functions and regulatory interactions of SA-responsive lncRNAs, thus forming the foundation for future functional analysis of SA-responsive lncRNAs in *Populus × euramericana*.

## 1. Introduction

Long noncoding RNAs (lncRNAs), refers to transcripts that have a length of more than 200 nucleotides (nt) and contain no apparent coding sequence (CDS). lncRNAs can modulate gene expression on various levels, through which biological pathways are finely tuned in plants, to respond to stress and adapt to adverse conditions. Generally, they regulate the transcriptional level of the target loci through *cis*-action or *trans*-action, showing obvious tissue-specific expression patterns and responses to environmental change [1]. In tomato, lncRNA–mRNA networks have been established, and lncRNA16397 was identified as modulating *SlGRX* expression, to reduce reactive oxygen species (ROS) accumulation, thus improving disease resistance [2]. Therefore, research on lncRNAs and coexpressed mRNAs could uncover the regulatory mechanisms of biological processes in plants from a new angle. 

Poplar, one of the most widely planted tree species in the world, has important economic value. *Populus* × *euramericana* is the first choice tree species for establishing fast-growing and high-yielding forests, as well as river protection forests, because of its excellent characteristics, such as rapid growth, hardiness, and water resistance. However, various stresses in the natural environment seriously restrict its production and application.

The chemical component of salicylic acid (SA) is o-hydroxybenzoic acid, which is a small-molecule phenolic substance, that is ubiquitous in plants. John Buchner isolated salicyl glycoside from willow bark in 1828, which initiated research on salicylic acid. Salicylic acid is considered a type of plant hormone, a signal molecule of plant stress response, that plays an important role in plant resistance [3]. It has remarkable effects on plant disease resistance, salt tolerance, seed germination, plant development, endogenous signal transmission, and pathogen defense [4]. Studies have also shown that exogenous SA can induce changes in the activities of protective enzymes (phenylalanine ammonia lyase, polyphenol oxidase, and so forth), which are closely related to plant resistance [5]. Spraying exogenous SA solution on the leaves of rice seedlings reduces the incidence of rice blast after spraying, indicating that SA, as an exogenous hormone, can induce rice plant defenses [6]. Spraying methyl salicylate solution on the leaves of rice seedlings can also enhance the stress resistance of rice [7]. SA is effective in inhibiting *P. expansum*-induced blue mold in apple, peach, and citrus fruits [8]. In the past decade, several studies have identified SA-responsive genes, making significant progress in understanding the response to SA. Such as, *PeTGA1*-overexpression poplars showed elevated SA levels, thereby resulting in increased resistance to *C. gloeosporioides* [9]. However, there has been no genome-wide identification of SA-responsive lncRNAs in plants. As lncRNAs play important roles in regulating gene expression, the identification of SA-responsive lncRNAs could help us comprehensively understand the response to SAs, and will provide important resources for the further functional analysis of these lncRNAs. 

Therefore, studying the role and mechanism of SA in plant stress responses has great theoretical and practical significance. In this study, the leaves of high-quality clones of *Populus* × *euramericana* were treated with H_2_O and SA, respectively, and the changes in phenylalanine ammonia-lyase (PAL) and polyphenol oxidase (PPO) activities in the leaves, under the different treatments, were determined. High-throughput RNA sequencing (RNA-seq) was used to identify messenger RNA (mRNA) and lncRNA, and the gene expression patterns in the SA-treated and control leaves were analyzed. Based on the *cis*-action or *trans*-action of lncRNA, the interaction between differentially expressed genes (DEGs) and lncRNAs (DELs) was analyzed, to identify the roles of lncRNAs in regulating gene expression and reveal the roles of lncRNAs in response to SA stimulation in *Populus* × *euramericana*. Overall, the results in this study increase our understanding of SA-responsive lncRNAs in *Populus* × *euramericana* and provide a rich resource for further research on the functions of these SA-responsive lncRNAs.

## 2. Results

### 2.1. Induction Effect of Exogenous SA on PAL and PPO Activities in Leaves of Populus × euramericana

**Figure 1:**
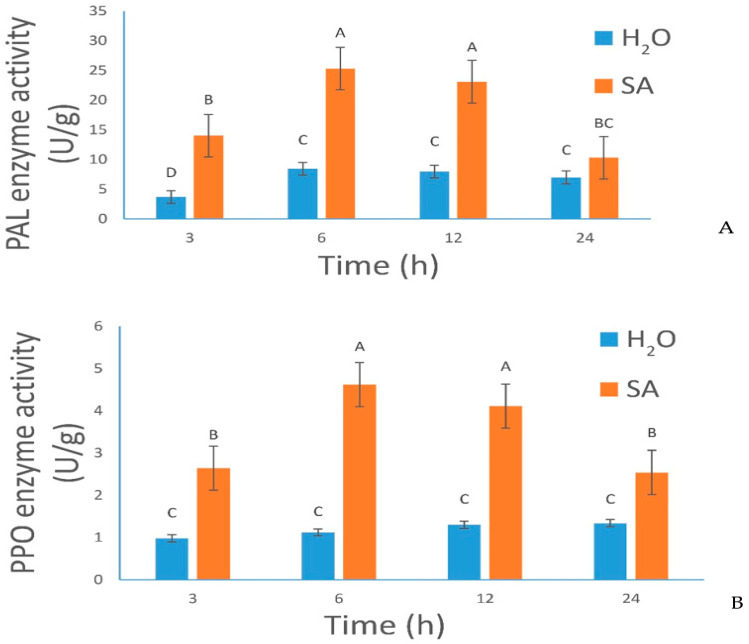
Effects of SA treatment on enzymatic activity. (**A**) and (**B**): Changes in phenylalanine ammonia-lyase (PAL) and polyphenol oxidase (PPO) activity in leaves at 3, 6, 12, and 24 h after treatments, respectively. Error bars indicate SDs among three biological replicates (*n* = 3). Different letters indicate significant differences between SA-treated and untreated control samples. H_2_O, control samples; SA, SA-treated samples.

As shown by Figure 1, the activities of PAL and PPO in leaves of *Populus* × *euramericana* changed greatly after spraying with H_2_O and SA. The activity of PAL and PPO in the SA-treated group first increased and then decreased, through 3, 6, 12, and 24 h, and the enzyme activity was higher in the SA group than in the control group at each time period (*p <* 0.05). However, the trend in the control group was not obvious. The activity of PAL and PPO peaked at 6 h. 

### 2.2. Genome-Wide Identification of mRNA and lncRNA in Leaves of Populus × euramericana Treated with SA

The activity of PAL and PPO peaked at 6 h. To obtain a comprehensive profile of the RNA expression, three biological repeats, at 6 h, of the control group (CK) and SA-treated group (T6H) were used for RNA-seq, resulting in six strand-specific libraries (CK-1, CK-2, CK-3, T6H-1, T6H-2, T6H-3). As shown in Table 1, for each replicate, over 78,992,406 raw sequence reads were generated. After raw data trimming, more than 78,794,420 clean reads were generated. The percentage of the GC content in the control group’s leaves was slightly higher than in the SA-treated group. Then, the ribosomal RNA (rRNA) was removed, and nearly 80.08% of valid reads were mapped to the *Populus trichocarpa* genome (assembling Pop_tri_v3) (https://www.ncbi.nlm.nih.gov/genome/98, accessed on 1 April 2022) for each replicate. The value of the Q20 proportion for all replicates was more than 96.86% (Table 1), indicating that the RNA-seq data were highly reliable.

**Figure 2:**
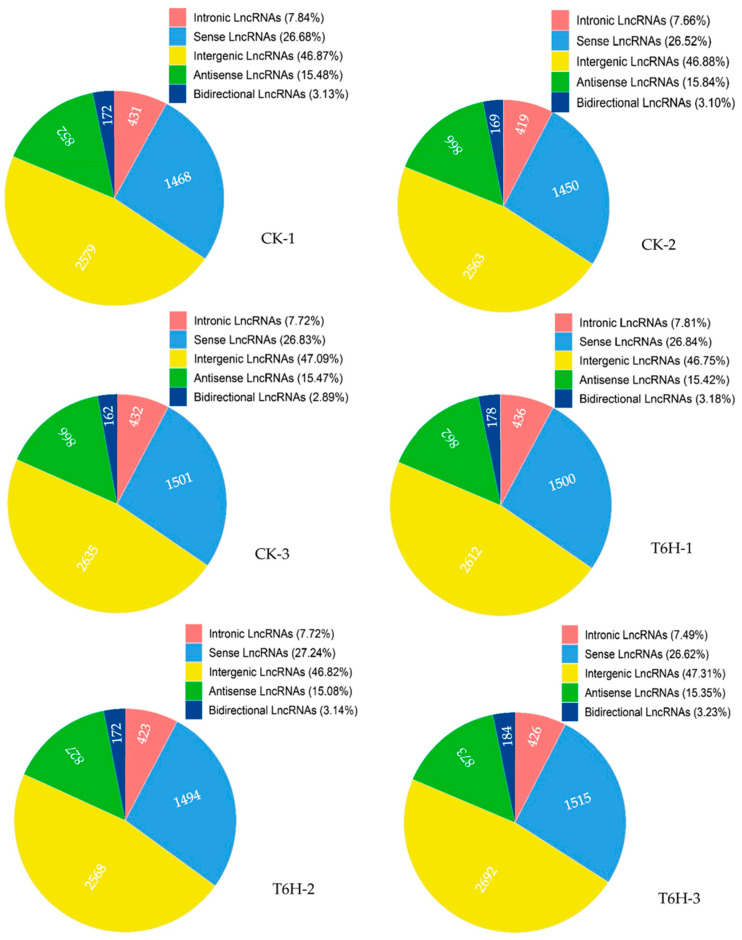
The proportion of the different types of lncRNAs in the six libraries. CK, control samples; T6H, SA-treated samples. The number in the pie chart represents the number of lncRNAs.

After the sequences were assembled, a total of 26,366 genes were obtained from the six libraries (Supplementary Table S1). Correspondingly, a total of 5690 lncRNAs were generated through the screening process (Table 2; Supplementary Table S2). The regulatory effect of a lncRNA is usually associated with its position relative to a protein-coding gene [10]. Accordingly, the lncRNAs identified in the SA-treated and control leaves were divided into five categories: intergenic lncRNA (yellow), intronic lncRNA (red), bidirectional lncRNA (dark blue), sense lncRNA (light blue), and antisense lncRNA (green). Figure 2 shows that intergenic lncRNA was the largest component, accounting for almost half of all lncRNAs in the SA-treated and control leaves.

### 2.3. Identification of Differentially Expressed Genes and lncRNAs

**Figure 3:**
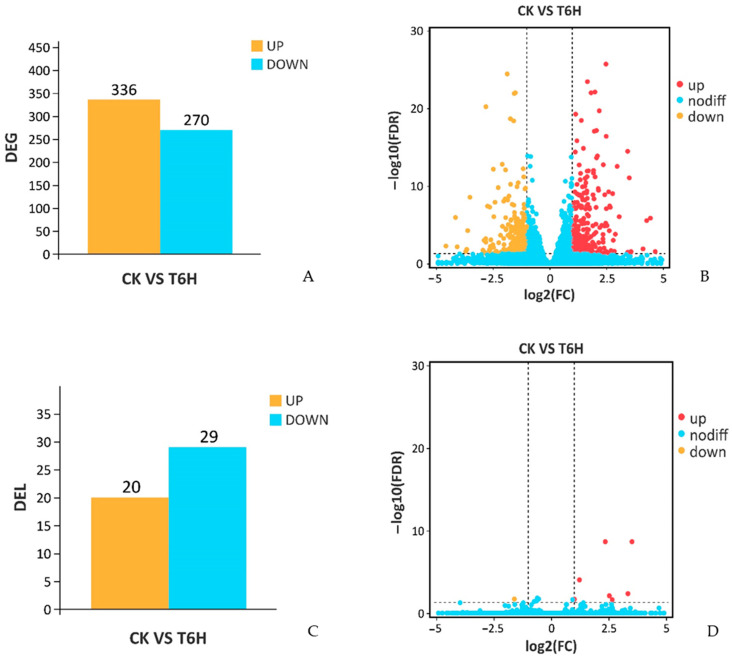
Comparison of differentially expressed genes (DEGs) and lncRNAs (DELs). (**A**) The number of DEGs, (**B**) the distribution of DEGs, (**C**) the number of DELs, (**D**) the distribution of DELs. CK, control samples; T6H, SA-treated samples.

To investigate the differences in the expression level of genes and lncRNAs between the SA-treated and control leaves, the fragments per kilobase per million mapped reads (FPKM) method was used to measure the expression level. Genes or lncRNAs with |log2 (fold change)| ≥ 1, and with statistical significance (*p*-value < 0.05), were considered as differentially expressed genes (DEGs) or differentially expressed lncRNAs (DELs). By comparing the leaves of the control group and SA-treated group, 606 DEGs, including 336 upregulated DEGs and 270 downregulated DEGs, were discovered (Figure 3A,B; Supplementary Table S3). Moreover, 20 upregulated and 29 downregulated DELs were found in the comparison (Figure 3C,D; Supplementary Table S4), so the total number of DELs was less than the total number of DEGs. 

**Figure 4:**
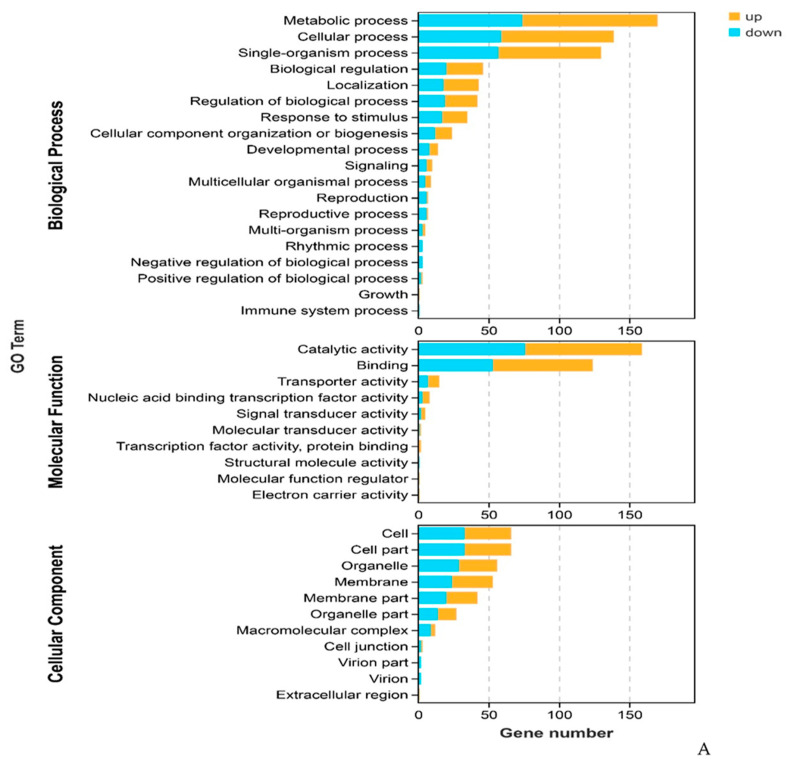

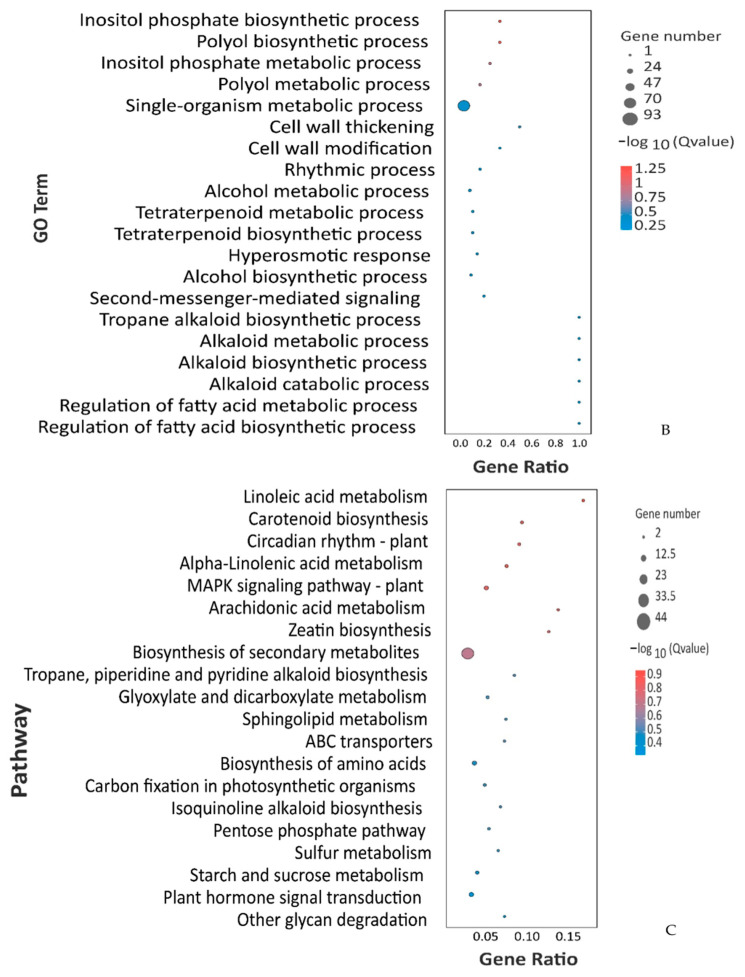
Characteristics of GO and KEGG pathways of differentially expressed genes (DEGs). (**A**) GO annotation, (**B**,**C**) GO terms and KEGG pathways (top 20) are both based on false discovery rate (FDR < 0.05).

GO annotation was further applied, to evaluate the functions of the DEGs, which were classified into “biological process”, “cellular component”, and “molecular function” categories (Figure 4A). Thus, to decipher the biological processes involved in stress response, GO enrichment of DEGs was performed. The results of the enrichment analysis of the GO annotation of DEGs, shown in Figure 4B, revealed that DEGs were mainly significantly enriched in “response to stimulus”, “regulation of biological process”, and “single-organism process” GO terms (Supplementary Table S5). Furthermore, the results of an enrichment analysis of KEGG pathways showed that DEGs were primarily significantly enriched in, the “metabolic pathways”, “biosynthesis of secondary metabolites”, “plant hormone signaling transduction”, “plant–pathogen interaction”, and “MAPK signaling pathway” categories (Figure 4C; Supplementary Table S6). These results indicate that there were DEGs between the SA-treated group and the control group, which were mainly involved in the secondary metabolism, hormone signal transduction, MAPK signaling, and plant–pathogen interaction pathways, in the stress response in *Populus* × *euramericana*.

### 2.4. Identification of Differentially Expressed lncRNA–mRNA Interaction Pairs

To determine the function of differentially expressed lncRNAs and their potential target mRNAs, interaction pairs of lncRNAs and mRNAs were predicted, based on *cis*- and *trans*-acting regulation patterns. The results showed that 259 differentially expressed mRNAs were identified as potential targets for the 36 differentially expressed lncRNAs in the SA-treated leaves. Then, based on the enrichment analysis of the GO annotation, we identified a number of target genes associated with “response to stimulus” and “regulation of biological process”, which are essential for regulation of plant growth and environmental response. Furthermore, by performing the enrichment analysis of the KEGG pathway, a number of target genes related to photosynthesis, signal transduction, plant circadian rhythm, and particularly plant–pathogen interaction, were identified in the SA-treated leaves, which may play important roles in responding to stress. These results demonstrate, that the differentially expressed lncRNA–mRNA interaction pairs may participate in photosynthesis, plant circadian rhythm, and plant–pathogen interaction pathways, which could be affected by exogenous SA, to respond to stress.

### 2.5. Interaction of lncRNAs and Target Genes Response to SA Stimulation of Populus × euramericana Leaves

**Figure 5:**
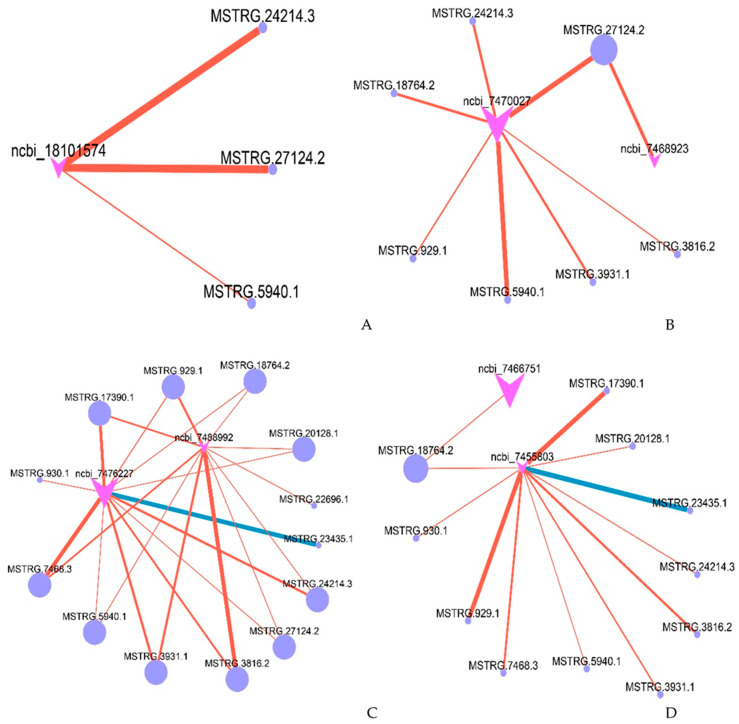
Prediction of DEGs and DELs related to light adaptation (**A**), abiotic stress (**B**), disease resistance (**C**), and growth and development of plants (**D**). Purple dots represent lncRNA. Pink arrows represent target genes. Blue and red lines represent positive and negative correlations, respectively, between the expression levels of lncRNAs and target genes. The line width indicates the degree of difference.

LncRNAs could be involved in the response to stress by regulating their potential target genes. Based on the functional enrichment of target genes described above, we focused on the differentially expressed target genes related to light, abiotic stress response, biotic stress response, and growth and development functional clusters. Furthermore, in order to visualize the interaction relationships, networks were constructed using the Cytoscape software (Figure 5). Among the interactions, most of the potential target genes were regulated by several lncRNAs, while very few genes were merely potentially regulated by one lncRNA. 

We analyzed the lncRNA–mRNA interaction pairs involved in the light response. In this functional cluster, the differential gene ncbi_18101574 was obviously upregulated. It was predicted as the target gene of three DELs (*MSTRG.24214.3*, *MSTRG.27124.2*, and *MSTRG.5940.1*), and their expression changes in the interaction cluster showed negative correlations (Figure 5A).

As fixed organisms, plants have evolved accurate and complex stress response mechanisms, to adapt to different living environments. In this abiotic stress response cluster, two genes related to the abiotic stress response were obviously upregulated, namely *fructose-diphosphate aldolase 1* (ncbi_7470027) and *cytokinin dehydrogenase 1* (ncbi_7468923). *Fructose diphosphate aldolase 1* was predicted as the target gene of seven DELs (*MSTRG.18764.2*, *MSTRG.24214.3*, *MSTRG.27124.2*, *MSTRG.3816.2*, *MSTRG.3931.1*, *MSTRG.5940.1*, *MSTRG.929.1*). The expression of these seven lncRNAs was downregulated. *Cytokinin dehydrogenase 1* was predicted as the target gene of *MSTRG.27124.2*. *MSTRG.27124.2* regulated the expression of both *fructose-diphosphate acetal 1* and *cytokinin dehydrogenase 1* (Figure 5B).

In their natural environment, plants are constantly exposed to various microbial communities, including pathogens such as fungi, oomycetes, viruses, bacteria, and nematodes. Therefore, plants have developed a multi-layer immune response system. Plants can enhance resistance in response to the stimulation of external pathogens and then activate the expression of pathogenesis-related (PR) proteins. Two DEGs, *RPM1* (ncbi_7476227) and *PR-1* (ncbi_7488992), were obviously upregulated. *RPM1* was predicted as the target gene of *MSTRG.17391.1*, *MSTRG.18764.2*, *MSTRG.20128.1*, *MSTRG.23435.1*, *MSTRG.24214.3*, *MSTRG.27124.2*, *MSTRG.3816.2*, *MSTRG.3931.1*, *MSTRG.5940.1*, *MSTRG.7468.3*, *MSTRG.929.1*, and *MSTRG.930.1*; and *PR-1* was predicted as the target gene of *MSTRG.17390.1*, *MSTRG.18764.2*, *MSTRG.20128.1*, *MSTRG.22696.1*, *MSTRG.24214.3*, *MSTRG.27124.2*, *MSTRG.3816.2*, *MSTRG.3931.1*, *MSTRG.5940.1*, *MSTRG.7468.3*, and *MSTRG.929.1*. Among these, *MSTRG.17390.1*, *MSTRG.18764.2*, *MSTRG.20128.1*, *MSTRG.24214.3*, *MSTRG.27124.2*, *MSTRG.3816.2*, *MSTRG.3931.1*, *MSTRG.5940.1*, *MSTRG.7468.3*, and *MSTRG.929.1* regulated the expression of both *RPM1* and *PR-1* (Figure 5C). Except for *MSTRG.23435.1*, the lncRNAs were negatively correlated with mRNA.

Genes related to plant hormones and circadian rhythms can regulate the growth and development of plants and may play an important role in the response of *Populus* × *euramericana* to SA stimulation. In this study, two DEGs, *LNK3* (ncbi_7455803) and *gibberellin 20 oxidase* (ncbi_7466751), were upregulated. *LNK3* was predicted as the target gene of *MSTRG.17390.1*, *MSTRG.18764.2*, *MSTRG.20128.1*, *MSTRG.23435.1*, *MSTRG.24214.3*, *MSTRG.3816.2*, *MSTRG.3931.1*, *MSTRG.5940.1*, *MSTRG.7468.3*, *MSTRG.929.1*, and *MSTRG.930.1*. *Gibberellin 20 oxidase* was predicted as the target gene of *MSTRG.18764.2*. *MSTRG.18764.2* regulated the expression of both *LNK3* and *gibberellin 20 oxidase* (Figure 5D). Except for *MSTRG.23435.1*, the lncRNAs were negatively correlated with mRNA.

### 2.6. Verification of qRT-PCR

**Figure 6:**
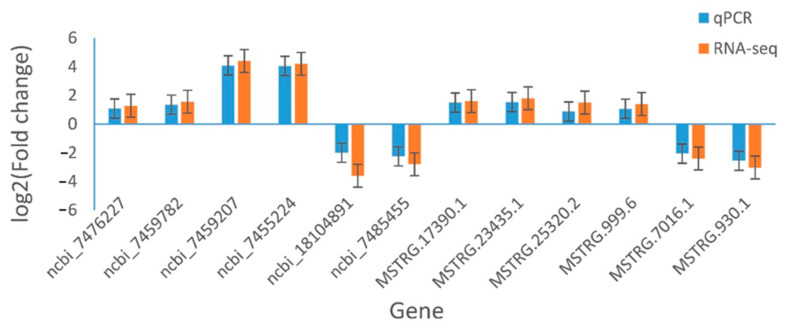
Relative expression of Genes. Error bars indicate SDs among three biological replicates (*n* = 3).

Based on functional analysis of target genes and lncRNAs, six DEGs and six DELs were randomly selected for qRT-PCR validation, using the same samples used for RNA-seq, to confirm the date of gene and lncRNA expression. The primers of DEGs and DELs are shown in Supplementary Table S7. Overall, the results showed that the expression profiles of the candidate genes and lncRNAs obtained from the qRT-PCR analysis were relatively consistent with those from the high-throughput RNA sequencing (Figure 6). These results indicate that the profile of gene and lncRNA expression from the high-throughput RNA sequencing is reliable, and they further confirm the differences in stress response in leaves with applied exogenous SA.

## 3. Discussion

### 3.1. Analysis of the Change Trend of Two Types of Main Defensive Enzyme Activities in Leaves Treated with SA

PPO exists ubiquitously in various plant tissues. It was considered to be a plant defense protein. It mainly oxidizes phenols in insect food into highly toxic quinones, to affect the growth and development of insects, slowing down the growth and development of insects or killing them. PAL is an enzyme that can use phenylalanine for the synthesis of phenolic compounds. When bacteria invade, the cells are stimulated to start the PAL system to produce lignin and deposit it around the cell wall, so as to limit the pathogen to a certain cell range and prevent it from further spreading. Most studies have shown that the activity of PAL has a highly positive correlation with the disease resistance of varieties [11]. Spraying exogenous SA can induce changes in PAL and PPO activities in plants, affect insect digestion, and enhance plant resistance, thus achieving insect resistance [11]. Liu et al. demonstrated that SA significantly induces PAL and PPO activities in the leaves of rice seedlings [12]. John Young and other researchers revealed that SA treatment can effectively induce insect resistance in cucumber [13]. Krajnc et al. suggested that exogenous SA is the activator of systemic acquired resistance in Norwegian spruce, and provides tolerance to the complex interaction between bark beetle attack and environmental factors [14]. In addition, the SA signaling pathway plays a role in regulating the indirect defense effect and physiological metabolism induced by *Bemisia tabaci* [15]. In this experiment, a 100 µM solution of SA was sprayed on *Populus* × *euramericana*, to induce a defense response. The results showed that spraying exogenous SA, significantly increased the activities of PAL and PPO in the leaves of *Populus* × *euramericana*. The change trends of PAL and PPO first increased and then decreased, and the activities of both, peaked at 6 h. The activities of PAL and PPO in the leaves did not change obviously after spraying with H_2_O. Liu et al. found that when salicylic acid was sprayed on the needles of *Larix kaempferi*, the activities of PAL and PPO in the leaves increased significantly, compared with those in the control group, and showed a fluctuating change, first rising and then falling, but there was no obvious change in the activities of PAL and PPO in the control group [11]. This result is consistent with our study. The results showed that exogenous SA could induce the expression and accumulation of two main defense enzymes in poplar, producing a positive effect on poplar’s ability to cope with stress.

### 3.2. Differential Expression Patterns of Genes and lncRNA in Leaves Treated with SA

Regulation of gene expression plays an important role in plant adaptation to the environment [16], and the expression patterns and regulatory functions of lncRNAs have attracted much attention. Some studies have shown that lncRNAs can regulate the expression of target genes involved in plant responses to the surrounding environment [17]. Our high-throughput RNA-seq results produced nearly 78,992,406 valid data from SA-treated leaves and controls (Table 2). The abundant and effective data enabled us to investigate the changes in mRNA and lncRNA expression in leaves, after application of exogenous SA. In total, 26,366 genes and 5690 lncRNAs were detected in *Populus* × *euramericana* (Table 2; Supplementary Tables S1 and S2). In addition, 606 DEGs and 49 DELs were identified in a comparison between leaves with exogenous SA application and control leaves (Figure 3A,C; Supplementary Tables S3 and S4). These DEGs and DELs may help to improve the stress resistance of the leaves.

### 3.3. LncRNA–mRNA Interaction in Photoreaction

Photosynthesis is essential to plants; however, when the light absorbed exceeds the photosynthetic capacity of plants, reactive oxygen species (ROS) are produced in chloroplasts, causing oxidative damage to proteins, lipids, and photosynthetic pigments. In addition, environmental pressures, such as low temperatures or drought, can inhibit the photosynthetic activity of crops, thus amplifying this effect and causing significant reductions in crop yield. Early light-induced proteins (ELIPs) belong to a family of polygenic light-trapping complexes that bind to chlorophyll in green plants and absorb solar energy. Studies have shown that ELIPs protect plant leaves from photooxidation, and that this photoprotection function involves maintaining low levels of free chlorophyll, under strong light [18]. ELIPs constitute the defense system of plants under light stress and may become a new selective marker for identifying and breeding crops with greater resistance to light oxidation stress. Barley varieties grown in northern Europe have more accumulated ELIPs than those grown in southern Europe [19]. We believe that the early photoinducible protein 1 found in this study, is involved in the photoprotection mechanism of *Populus* × *euramericana*, and that exogenous SA can improve the ability of *Populus* × *euramericana* to cope with photodamage.

### 3.4. LncRNA–mRNA Interaction in Abiotic Stress Response

To survive various unfavorable external environments, plants have evolved protective systems, which usually implement lncRNA–mRNA interactions [20]. Fructose-diphosphate aldolase (FBA) exists widely in bacteria, higher animals, plants, and other organisms, and many studies have shown that FBA responds to various biotic [21] and abiotic stresses [21,22]. For example, 12-day-old wheat seedlings had upregulated FBA activity under salt stress, which helped the seedlings adapt to the stress [23]. In chickpeas, FBA activity is inhibited under water stress [24]. Overexpression of the *SlFBA4* gene in tomato can promote growth and cold resistance [25]. Therefore, we believe that the fructose-diphosphate aldolase 1 (ncbi_7470027) gene found in this study, plays an important role in coping with abiotic stress in *Populus* × *euramericana*. 

Cytokinin (Cks), a plant hormone, participates in plant morphogenesis and also in the regulation of many physiological processes, including tolerance to drought stress [26,27]. Studies have shown that barley is transformed by the *Arabidopsis thaliana cytokinin dehydrogenase 1* gene (*AtCKX1*), under the control of the maize mild root-specific β-glucosidase promoter. The increase in cytokinin degradation activity has a positive effect on the number and length of lateral roots. Under severe drought stress, all transgenic varieties maintain high water content and show good growth and yield parameters during recovery. Therefore, we believe that the cytokinin dehydrogenase 1 found in this study, plays a role in coping with drought stress in *Populus* × *euramericana*.

### 3.5. LncRNA–Target Gene Interaction in Disease Resistance in Poplar Leaves

In the natural environment, plants are constantly exposed to various microbial communities, including pathogens such as fungi, oomycetes, viruses, bacteria, and nematodes. Therefore, plants have developed a multi-layer immune response system, including defense mechanisms, ranging from basic resistance to an induced resistance called innate immunity. Innate immunity is divided into two categories: pathogen-related molecular pattern (PAMP)-triggered immunity (PTI) and effector-triggered immunity (ETI) [28]. Both PTI and ETI can activate the expression of PR and induce defense signaling hormones [29,30], such as *RPM1*, *PR-1*, *RPS2*, and *RPS4*. 

RPM1 is a NOD-like receptor (NLR), an R gene product identified in *A. thaliana*, that confers resistance to *Pseudomonas syringae* expressing either of two effector proteins, avrRpm1 or avrB. Studies have shown that phosphorylated protein RPM1 occurs in resistant tomatoes, but not in susceptible tomatoes [31]. The plant defense hormone SA, is involved in the biological response to nutrient pathogens, and it can induce the expression of different R genes. SA treatment was shown to induce *TaRPM1* expression in wheat [32]. In addition, the wheat R gene *TaRGA*, was induced by exogenous application of SA, and endowed wheat with resistance to powdery mildew [32]. In tomato, *RPM1* may enhance resistance by enhancing the cell wall, through ROS production mediated by glycoprotein crosslinking. Therefore, we believe that the *RPM1* gene in this study improves the disease resistance of *Populus* × *euramericana*.

Since the discovery in 1970 of the PR-1 protein, many researchers have attempted to evaluate its function in plants, but with little success. The limited antifungal activity suggests a function in plant defense, but the mode of action and relationship with other proteins remain unclear [33]. In *A. thaliana*, expression of SA-dependent *PR-1*, *PR-2*, and *PR-5* is necessary, to enhance protection against parasitic frost mold [34]. Moreover, following inoculation of *Beauveria bassiana*, a large amount of SA accumulated in *Populus tomentosa* tissues, but the control did not induce SA accumulation under the same conditions, indicating that the change in SA was closely related to *B*. *bassiana* infection. Furthermore, the expression of the pathogenic genes *PR-1*, *PR-2*, and *PR-5* was upregulated under higher SA content [35]. Jiang found that SA can induce and enhance disease resistance in poplar [36]. Under the stresses of canker, skin rot, and leaf rust, poplar can activate the SA signal transduction pathway and significantly upregulate the expression of the PR protein gene, thus enhancing resistance [37,38]. Therefore, we believe that the *PR-1* gene in this study can improve the disease resistance of *Populus* × *euramericana*.

### 3.6. LncRNA–mRNA Interaction in Growth and Development

Light is both the energy source of plants and also an important environmental factor affecting their growth and development [39]. Light signals regulate the growth and development of plants, by affecting their circadian rhythm [40]. Optical signaling pathways interact with biological clocks, to help organisms synchronize physiological and developmental processes with periodic environmental cycles. Members of the luminous induction and clock regulation (LNK) gene family, play the key role in *A. thaliana,* linking the regulation of gene expression by light with the control of diurnal and seasonal rhythms. In particular, *LNK1* and *LNK2* have been shown to control circadian rhythms, photomorphogenesis responses, and photoperiod-related flowering times. Moreover, studies have shown that deletion of *LNK3* and *LNK4*, which are closely related in the background of *LNK1* and *LNK2* mutations, affects the circadian rhythm. As a result, plants show developmental changes, resulting in increased rosette size, increased biomass, and enhanced photoinduced response. Members of the LNK family are an important link in coordinating the development of the light regulation process [41]. Therefore, we believe that the *LNK3* in this study plays a role in regulating the circadian rhythm of *Populus* × *euramericana* and thus affects its growth and development.

Gibberellins (GAs) are important plant hormones, that regulate plant growth and development, including seed germination, flowering, stem elongation, and wood formation [42,43]. *GA20-oxidase* (*GA20ox*), which also plays a key role in plant growth and development, has been cloned from many plants [44,45], and its overexpression has been demonstrated to increase GA levels, resulting in accelerated stem growth in plants such as *A. thaliana* [46], potato [47], and hybrid poplar [48]. We believe that the *GA20ox* in this study plays an active role in the growth and development of *Populus* × *euramericana*.

Plant lncRNAs play roles in responses to biotic and abiotic stress [49,50,51]. For example, 125 lncRNAs involved in powdery mildew infection and high-temperature stress, have been identified in wheat [52]. After exposure of *A. thaliana* to drought, cold, high salt, or abscisic acid, the expression of 1832 lncRNAs was markedly upregulated, when compared with the control group [53]. More recently, genome-wide studies showed that lncRNAs respond to heat and salt stress in Chinese cabbage (*Brassica rapa*) and poplar (*Populus trichocarpa*), respectively [54,55]. *AtR8* lncRNA participates in *PR-1*-independent defense and root elongation, which are related to the SA response [56]. Liu et al. demonstrated that lncRNA *SABC1* acts as a molecular switch in balancing *A. thaliana* defense and growth, by modulating SA biosynthesis [57]. 

In summary, SA plays an important role in the biotic and abiotic stress resistance of *Populus* × *euramericana*. lncRNA–mRNA interactions, following exogenous SA application, were involved in the response of poplar leaves to the external environment, which adds to our understanding of the molecular mechanism of the response of poplar to stress. 

## 4. Materials and Methods

### 4.1. Plant Materials and SA Treatment

One-year-old *Populus* × *euramericana* plants were grown under 16 h of light and 8 h of dark, in a greenhouse at Hebei Agricultural University, Baoding, China (38°85′ N, 115°48′ E). For SA treatment, 100 µM SA was sprayed on seedling leaves until drops of liquid dripped down. The control plants were treated with H_2_O in the same manner. Mature leaves, from the same positions of control plants and SA-treated plants, were collected at 3, 6, 12, and 24 h after treatment, and three biological replicates were sampled per time point. All the harvested leaves were immediately frozen in liquid nitrogen after collection, and stored at −80 °C until use.

### 4.2. Determination of Enzyme Activity

The enzyme activity of *Populus* × *euramericana* was determined according to the instructions accompanying the PAL and PPO kits, purchased from Suzhou Keming Biotechnology Co., Ltd. (Suzhou, China).

### 4.3. RNA Isolation, Library Construction, and RNA Sequencing

We used leaves from the control and SA-treated plants 6 h after treatment (three biological replicates per treatment) for high-throughput RNA-seq. Total RNA was extracted using the Juhemei RNAeasy kit (Juhemei, Beijing, China), according to the manufacturer’s instructions. Additional on-column DNase digestions were performed during the RNA purification, using an RNase-Free DNase Set (Juhemei). The RNA samples were assessed with the NanoDrop ND-1000 spectrometer (Thermo Scientific, Waltham, MA, USA) and Agilent Bioanalyzer 2100 (Agilent, Santa Clara, CA, USA) before RNA-seq, and then used to construct strand-specific RNA-seq libraries, according to the TruSeq RNA Sample Preparation Guide. After being quantified with the fluorometer (Qubit 2.0, Thermo Scientific) and bioanalyzer (model 2100, Agilent), the strand-specific libraries were sequenced on an Illumina HiSeq^TM^ 4000 instrument (Illumina, San Diego, CA, USA). Library construction and Illumina sequencing were carried out by the Gene Denovo Biotechnology Corporation (Wuhan, China). The sequencing data have been submitted to the NCBI Sequence Read Archive (BioProject accession number PRJNA926326).

### 4.4. Identification of SA-Responsive mRNAs and lncRNAs 

Reads obtained from the sequencing machines include raw reads containing adapters or low-quality bases, which will affect the following assembly and analysis. Thus, to get high-quality clean reads, reads were further filtered by FASTQ (https://github.com/OpenGene/fastp, accessed on 1 April 2022). The parameters were as follows:

(1) removing reads containing adapters;
(2) removing reads containing more than 10% of unknown nucleotides (N);
(3) removing low-quality reads, containing more than 50% of low-quality (Q-value ≤ 20) bases.

The short reads alignment tool Bowtie2 (https://nchc.dl.sourceforge.net/project/bowtie-bio/bowtie2, accessed on 1 April 2022) was used for mapping reads to a ribosome RNA (rRNA) database. The rRNA mapped reads were then removed. The remaining clean reads were further used in assembly and gene abundance calculation. Then the clean reads were aligned to the *P. trichocarpa* genome (assembling Pop_tri_v3) (https://www.ncbi.nlm.nih.gov/genome/98, accessed on 1 April 2022) using Tophat v2.0.9 [58]. The reads with no more than three mismatches were used to assemble transcripts of each sample separately. The mapped reads of each sample were assembled by using StringTie v1.3.1, in a reference-based approach. For each transcription region, a FPKM (fragment per kilobase of transcript per million mapped reads) value was calculated, to quantify its expression abundance and variations, using the StringTie software [59]. The FPKM formula is as follows:

$$\mathrm{FPKM}=\frac{10^{6}C}{\mathrm{NL}/10^{3}}$$

Given FPKM(A) to be the expression of gene A, C to be number of fragments mapped to gene A, N to be total number of fragments that mapped to reference genes, and L to be number of bases on gene A. The FPKM method is able to eliminate the influence of different gene lengths and sequencing data amount, on the calculation of gene expression. Therefore, the calculated gene expression can be directly used for comparing the difference of gene expression among samples. The prediction of lncRNAs from RNA-seq data was performed according to Sun et al. [60]. Transcripts with mapping coverage of less than half the transcript length and transcripts with FPKM < 1, were removed. Any transcripts that were shorter than 200 bp, or encoded ORFs longer than 100 amino acids, were discarded. The coding potential of the remaining transcripts was evaluated using the coding potential calculator (CPC) software (http://cpc.cbi.pku.edu.cn/, accessed on 1 April 2022) and Coding Noncoding Index (CNCI) software (http://www.bioinfo.org/software/cnci, accessed on 1 April 2022). When using CPC, we used the protein-coding transcripts of *P. trichocarpa* as a reference. The lncRNAs were classified into intergenic, intronic, bidirectional, sense, and antisense lncRNAs, using the cuffcompare program in the Cufflinks suite [59,60]. The expression level for mRNAs and lncRNAs was represented as the fold change (FC) = FPKM of CK/FPKM of T6H. Only the mRNAs and lncRNAs that met the criteria of |log2 (fold change)| ≥1, and with statistical significance (*p* < 0.05), were considered SA responsive.

### 4.5. Target Gene Prediction of lncRNAs and Establishment of Coexpression Networks

The potential target genes of SA-responsive lncRNAs were predicted according to their regulatory effects, which were divided into *cis*- and *trans*-acting. Two independent algorithms were used. The first algorithm searches for potential *cis*-target genes that are physically close to lncRNAs (within 10 kb), by using genome annotation and a genome browser. The genes transcribed within a 10 kb window upstream or downstream of lncRNAs, were considered potential *cis*-target genes [61]. The second algorithm searches for potential *trans*-targets in the Populus mRNA database, and is based on mRNA sequence complementarity and RNA duplex energy prediction, assessing the impact of lncRNA binding on complete mRNA molecules. First, we used BLAST to select target sequences that were complementary to the lncRNA, setting the E-value < 1e-5 and identity ≥ 95%. Then we used the RNAplex software to calculate the complementary energy between two sequences for further screening, and to select potential *trans*-acting target genes (RNAplex -e-60) [62]. To visualize the interaction between lncRNAs and target protein-coding genes, the Cytoscape software was used to establish the networks of lncRNAs and target genes [63].

### 4.6. Gene Ontology and Pathway Enrichment Analysis

Before GO and pathway enrichment analysis, the predicted target genes were annotated by PopGenie (http://www.popgenie.org/, accessed on 1 April 2022) [64]. Then GO terms were identified with AgriGO (http://bioinfo.cau.edu.cn/agriGO/index.php, accessed on 1 April 2022), using suggested backgrounds [65], and the permutated false discovery rate (FDR) value cut-off was set at 0.05. The enrichment pathway analysis was conducted to analyze the potential functions of the target genes in the pathways, by using the KEGG (Kyoto Encyclopedia of Genes and Genomes) database (http://www.genome.ad.jp/kegg/, accessed on 1 April 2022) and a hypergeometric statistical test [66,67].

### 4.7. qRT-PCR Validation of mRNA and lncRNA Expression Levels

Total RNAs obtained from the leaves of the control and SA-treated plants at 6 h after treatment, were reverse transcribed into cDNA and used for measuring the expression of SA-responsive mRNAs and lncRNAs by real-time quantitative polymerase chain reaction (qRT-PCR). Total RNA was extracted using the plant RNA Kit (Juhemei, Beijing, China). Then the sequence was reverse transcribed into cDNA using the “chiliu” reverse transcription kit (Juhemei, Beijing, China). Six DEGs and six DELs were randomly selected for verification. Then, the Primer 5.0 software (Premiere Biosoft, San Francisco, CA, USA) was adopted to design the gene-specific primers. The qRT-PCR reaction was performed with the MiniOpticon Two-Color Real-Time PCR Detection System (Bio-Rad, Hercules, CA, USA), using SuperReal PreMix Plus (Tiangen, Beijing, China). All reactions were carried out with three replicates, following two-step cycling conditions: 95 °C for 10 min, then 45 cycles at 95 °C for 10 s and 60 °C for 30 s. The generated real-time data were analyzed using the Opticon Monitor Analysis Software 3.1 tool and standardized to the levels of poplar ACTINII-like (accession number EF145577), using the ΔΔCT method (fold change = 2^−ΔΔCT^) [68]. The primers used for qRT-PCR are listed in Supplementary Table S7.

## 5. Conclusions

In this study, we analyzed lncRNA expression profiles and regulation of target genes in SA-treated leaves, which were from *Populus × euramericana.* Our findings showed that the activities of PAL and PPO in the leaves of *Populus × euramericana* were significantly increased by exogenous SA application. The interactions of candidate DELs and target DEGs associated with the response to the external environment were determined, suggesting a greater tolerance to adverse environmental conditions of SA-treated leaves. Some lncRNAs (such as *MSTRG.24214.3*, *MSTRG.27124.2*, *MSTRG.5940.1 and MSTRG.23435.1*) and mRNAs (such as *RPM1, PR-1, LNK3 and GA20ox*), were found to respond positively to the external environment by participating in the light response, stress response, plant disease resistance, and growth and development pathway, which adds to our understanding of the molecular mechanism of the response of poplar to stress. This study provides a comprehensive view of *Populus × euramericana* lncRNAs and offers insights into the potential functions and regulatory interactions of SA-responsive lncRNAs, thus forming the foundation for future functional analysis of SA-responsive lncRNAs in *Populus × euramericana*.

## Figures and Tables

**Table 1:** Statistical data of RNA-seq reads for the six libraries constructed from leaves treated with H_2_O and SA.

Sample	RawData	CleanData	Q20 (%)	Q30 (%)	GC (%)
CK-1	83,112,030	82,892,416	98.13%	94.24%	43.53%
CK-2	79,791,830	79,577,780	98.13%	94.26%	43.76%
CK-3	78,992,406	78,794,420	98.08%	94.08%	43.07%
T6H-1	80,777,364	80,595,986	98.08%	94.10%	42.99%
T6H-2	84,664,040	844,695,186	96.86%	91.13%	43.00%
T6H-3	115,762,366	11,563,076	97.69%	93.07%	42.92%

Notes: CK, control samples; T6H, SA-treated samples. Q20% and Q30%, proportions of the data for which quality values were greater than Q20 and Q30 in the raw data, respectively.

**Table 2:** Statistics of genes and lncRNA expressed in six libraries constructed from leaves treated with H_2_O and SA.

Sample	CK-1	CK-2	CK-3	T6H-1	T6H-2	T6H-3
Expressed gene	26,118	25,759	26,366	25,423	25,869	25,846
Expressed lncRNA	5502	5467	5595	5587	5485	5690

Notes: CK, control samples; T6H, SA-treated samples.

## Data Availability

The data presented in this study are openly available in the NCBI Sequence Read Archive (BioProject accession number PRJNA926326).

## Supplementary Materials

The following supporting information can be downloaded at: https://www.mdpi.com/article/10.3390/plants12061377/s1, Table S1: Summary of *Populus × euramericana* gene data; Table S2: Summary of Populus × euramericana lncRNA data; Table S3: Summary of differentially expressed genes; Table S4: Summary of differentially expressed lncRNAs; Table S5: The significant GOs of DEGs; Table S6: The significant pathways of DEGs; Table S7: Primers used for real-time quantitative PCR.
